# 46,XY DSD with Partial Gonadal Dysgenesis and Growth Failure in a Patient with 3q27.1 Microdeletion: Candidate Gene Curation After Exhaustive Literature Review

**DOI:** 10.3390/ijms27020821

**Published:** 2026-01-14

**Authors:** Lourdes Correa Brito, Sofía Suco, Bárbara Casali, Florencia Villegas, Paula Scaglia, Agustín Izquierdo, Jimena Lopez Dacal, Miguel Podestá, Martín Medin, Romina P. Grinspon, María Gabriela Ropelato, Rodolfo A. Rey

**Affiliations:** 1 Centro de Investigaciones Endocrinológicas “Dr. César Bergadá” (CEDIE), CONICET-FEI-División de Endocrinología, Hospital de Niños Ricardo Gutiérrez, Buenos Aires C1425EFD, Argentina; lcorrea@cedie.org.ar (L.C.B.); ssuco@cedie.org.ar (S.S.); bcasali@cedie.org.ar (B.C.); pscaglia@cedie.org.ar (P.S.); aizquierdo@cedie.org.ar (A.I.); jlopezdacal@cedie.org.ar (J.L.D.); rgrinspon@cedie.org.ar (R.P.G.); gropelato@cedie.org.ar (M.G.R.); 2 Unidad de Medicina Traslacional, Hospital de Niños Ricardo Gutiérrez, Buenos Aires C1425EFD, Argentina; 3 Sección de Genética Médica, Hospital de Niños Ricardo Gutiérrez, Buenos Aires C1425EFD, Argentina; florenciaevillegas@gmail.com; 4 Unidad de Urología, Hospital de Niños Ricardo Gutiérrez, Buenos Aires C1425EFD, Argentina; milito76@hotmail.com; 5 Servicio de Anatomía Patológica, Hospital de Niños Ricardo Gutiérrez, Buenos Aires C1425EFD, Argentina; martinmed797@hotmail.com

**Keywords:** 3q27.1 microdeletion, copy number variant (CNV), disorders of sex development (DSD), growth failure, genotype–phenotype correlation, small for gestational age (SGA)

## Abstract

Complex genetic syndromes represent a diagnostic challenge due to their diverse phenotypic presentations, which often evolve over time and may not be fully evident at birth. Disorders of sex development (DSD) comprise congenital conditions with discordance between chromosomal, gonadal, and/or genital sex. In 46,XY gonadal dysgenesis, undervirilisation or female-appearing genitalia may occur despite a normal karyotype, and diagnosis increasingly relies on genomic approaches. Prenatal and postnatal growth failure has been described in patients with syndromic 46,XY DSD. We report a male patient with SGA, lack of postnatal catch-up growth, and syndromic dysgenetic 46,XY DSD followed longitudinally from infancy to 11 years, in whom whole-exome sequencing (WES) reanalysis revealed a pathogenic 2.7 Mb microdeletion at 3q27.1q27.2. Systematic review of previously reported 3q27.1 deletions identified overlapping phenotypes but limited documentation of gonadal dysfunction. Curation of 71 genes within the deleted region highlighted *DVL3* and *CLCN2* as potential contributors to the gonadal phenotype, although functional evidence remains lacking. This case expands the phenotypic spectrum of 3q27.1 microdeletion syndrome, suggesting that 46,XY gonadal dysgenesis may represent an under-recognised feature. It also underscores the importance of copy number variant (CNV) analysis and periodic re-evaluation of sequencing data to increase diagnostic yield.

## 1. Introduction

Disorders of sex development (DSD) refer to a group of congenital conditions in which there is a discordance between chromosomal, gonadal, and/or genital sex. In 46,XY gonadal dysgenesis, a subtype of DSD, the 46,XY karyotype is normal, but the gonads and/or internal or external sex organs do not appear typically male; for instance, the external genitalia may appear ambiguous, i.e., have different degrees of undervirilisation, or even typically female [1]. 46,XY gonadal dysgenesis can present in isolation or as part of a syndrome, where the underlying genetic cause contributes to a broader phenotype involving multiple organ systems [2]. These complex genetic syndromes present a significant challenge in diagnostic approaches because the range of symptoms and signs may not be fully apparent at birth and emerge over time as the patient grows older.

Short stature is defined as a height below −2 standard deviations (SD) from the population mean for age and sex. Stature below −2 SD below mid-parental height, decreased growth velocity, and a deflection in the growth curve are anthropometric signs that indicate a higher probability of pathological conditions affecting growth [3]. Growth failure can be classified according to its origin, as prenatal or postnatal. The term “Small for Gestational Age” (SGA) describes children born with low birth height and/or weight, some of whom may experience a failure to catch-up on growth in postnatal life and present with short stature [4]. A strong association has been described between being born SGA and undervirilisation of 46,XY newborns, and many of these individuals with 46,XY DSD experience insufficient catch-up growth [5].

The existence of ambiguous genitalia in newborns SGA prompts referral to a specialist, which leads to a diagnostic workup, particularly including genetic testing. Next-generation sequencing (NGS) has become a frequently used technique due to its relatively high diagnostic rate [6,7]. When the initial NGS assessment results are negative, reanalysis of sequencing data has been shown to improve the diagnostic yield if advances in analytical capacity (including bioinformatic tools, filtering and prioritisation strategies, and coverage) are incorporated, together with an updated clinical characterisation of the patient’s phenotype [8].

Here, we present the case of a patient with syndromic 46,XY DSD born SGA and insufficient catch-up growth during long-term follow-up, in whom data of whole-exome sequencing (WES) reanalysis enabled a genetic diagnosis. In addition, we provide a detailed description of previously reported cases with the non-recurrent 3q27.1 microdeletion, together with a curation of the potential genes located within the patient’s deletion that may contribute to the DSD phenotype and growth failure.

## 2. Case Description

### 2.1. Patient and Methods

#### 2.1.1. Patient

The patient reported herein was evaluated at the Ricardo Gutiérrez Children’s Hospital (Buenos Aires, Argentina). Written informed consent for genetic diagnostics and for publication of clinical data and genetic results was obtained from the patient’s parents. The genetic assessment protocol including NGS was approved by the Institutional Review Board (Comité de Ética en Investigación del Hospital de Niños Ricardo Gutiérrez (reference number CEI 17.36, approval date 18 August 2020)).

#### 2.1.2. Clinical Assessment and Diagnostic Testing

Birth weight and height for gestational age were calculated as a standard deviation score (SDS) according to the INTERGROWTH-21st standards [9]. Length, measured using an infantometer, and weight, determined with a calibrated scale, were expressed as SDS based on the Argentine population references [10]. Physical examination performed by a paediatric endocrinologist included the assessment of external genitalia according to the External Genitalia Score (EGS) [11]. Serum follicle-stimulating hormone (FSH), luteinising hormone (LH), testosterone, 17-hydroxyprogesterone, androstenedione, oestradiol, and anti-Müllerian hormone (AMH) were measured using validated assays, as previously published [12,13,14]. Ultrasonography and cystourethroscopy were performed by a paediatric radiologist and a paediatric urologist, respectively. Further assessments were conducted by a neurologist, ophthalmologist, cardiologist, and geneticist at the same paediatric hospital.

#### 2.1.3. Genetic Analysis

Peripheral blood karyotype was performed using high-resolution G-bands by trypsin using Giemsa (GTG-banding). The genomic deoxyribonucleic acid (DNA) was extracted from peripheral venous blood cells using the Gentra Puregene Blood Kit (Qiagen, Hilden, Germany). The DNA was quantified using a high-performance microvolume spectrophotometer Nanophotometer^®^ NP60 (Implen Inc., Westlake Village, CA, USA). Whole-exome sequencing (WES) was performed by 3 Billion, Inc. (Seoul, Republic of Korea). All exon regions of all human genes (~22,000) were captured by xGen Exome Research Panel v2 (Integrated DNA Technologies, Coralville, IA, USA). The captured regions of the genome were sequenced with a Novaseq 6000 (Illumina, San Diego, CA, USA). Results were informed using 3 Billion’s standard procedures in 2022.

In the reanalysis of sequencing data in 2025, we followed the best practice recommendations from the Broad Institute using the Genome Analysis Toolkit (GATK) for preprocessing, variant calling, and refinement. Raw sequence data were mapped to the 1000-Genomes phase II reference genome (GRCh38 version GCF_000001405.40) using the BWA-MEM algorithm of Bur-rows-Wheeler Aligner software, version 0.7.15-r1140. Duplicates were removed using Picard (Broad Institute, Boston, MA, USA). The variant call format file (VCF) was annotated using ANNOVAR, date 2020-06-08 [15]. The horizontal coverage of a set of candidate genes associated with DSD, as well as genes identified through a preliminary search using the PHENOMYZER application, was assessed. Variant filtering and prioritisation were performed using Franklin by Genoox (https://franklin.genoox.com/, accessed on 13 February 2025). Candidate variants were selected following different strategies: (i) selection based on Human Phenotype Ontology (HPO) terms (Small for gestational age HP:0001518; Intrauterine growth retardation HP:0001511; Ambiguous genitalia HP:0000062; Penoscrotal hypospadias HP:0000808; Micropenis HP:0000054; Bilateral cryptorchidism HP:0008689; Low-set ears HP:0000369; Retrognathia HP:0000278; Clinodactyly of the 5th finger HP:0004209; 2–3 toe syndactyly HP:0004691; Patent foramen ovale HP:0001655; Specific learning disability HP:0001328; Attention deficit hyperactivity disorder HP:0007018; Esodeviation HP:0020045; Myopia HP:0000545; Dysphagia HP:0002015; Recurrent respiratory infections HP:0002205); (ii) filtering based on high-quality variants (depth >10×, GQ > 45) with a minor allele frequency (MAF) < 3% in gnomAD (https://gnomad.broadinstitute.org/ accessed on 13 February 2025), including high- and moderate-impact variants (frameshift, nonsense, missense, and splice site) in genes related to gonadal dysgenesis (ARX, ATRX, CBX2, DHH, DHX37, DMRT1, DMRT2, EMX2, ESR2, FGFR2, GATA4, HHAT, MAMLD1, MAP3K1, MYRF, NR0B1, NR5A1, PPP2R3C, SAMD9, SOX9, SRY, TSPYL1, WNT4, WT1, WWOX, ZFPM2 and ZNRF3) and (iii) prioritisation using the VarElect tool (https://varelect.genecards.org/, accessed on 13 February 2025), which scores genes obtained from an initial filtering step that considers only variants with MAF < 3% and high-quality metrics, based on their relevance to the case phenotypes defined by HPO terms. Integrative Genomics Viewer (IGV v.1.4.2) [16] was used to visually inspect the variants. Human Genome Variation Society (HGVS) nomenclature was checked with Mutalyzer 3 [17]. We classified the variants according to their potential pathogenicity using the American College of Medical Genetics and Genomics/Association for Molecular Pathology (ACMG/AMP) guidelines for variant interpretation [18] and following the ClinGen Sequence Variant Interpretation Working Group (SVI WG) recommendations (https://www.clinicalgenome.org/working-groups/sequence-variant-interpretation/, accessed on 16 February 2025). Applying the CNV prediction tool from NGS-derived data, DECoN (Detection of Exon Copy Number variants), we screened for potential CNV-type variants [19]. Additionally, array comparative genomic hybridisation (array-CGH) was performed to confirm CNV findings using SurePrint G3 ISCA V2 CGH 8x60K, G3 Unrestricted CGH (Agilent Technologies Inc., Santa Clara, CA, USA). Data processing and bioinformatic analysis were performed using Cytogenomics 5.2.14 software and the Aberration Detection Method 2 (ADM-2) algorithm (Agilent Technologies Inc., Santa Clara, CA, USA). CNVs were classified into 5 categories according to their degree of pathogenicity, following international standards [20].

#### 2.1.4. Systematic Literature and Database Review of Reported 3q27.1 Microdeletions

We performed a systematic review of previously reported cases with microdeletions involving chromosome band 3q27.1. The search strategy included three sources: (1) the DECIPHER database (https://www.deciphergenomics.org/, accessed on 31 October 2025), (2) ClinVar (https://www.ncbi.nlm.nih.gov/clinvar/, accessed on 31 October 2025), and (3) PubMed (https://pubmed.ncbi.nlm.nih.gov/). For PubMed, we used the search terms “3q27” and “deletion” or “microdeletion” and selected only reports in which the deletion involved 3q27.1. Articles published up to 31 October 2025 were included. From all identified reports, clinical and genetic data were extracted, with special emphasis on genital anomalies and gonadal function.

#### 2.1.5. Characterisation and Prioritisation of Candidate Genes Within Patient’s 3q27.1 Deletion

To systematically evaluate the 3q27.1 microdeletion in our patient, we compiled a list of 71 genes based on the DECIPHER database (https://www.deciphergenomics.org/). The evaluation of genetic and experimental evidence for each gene was guided by the framework proposed by the Clinical Genome Resource (ClinGen), which provides a systematic approach to assess the strength of gene–disease associations [21]. Gene annotations included gene name, HGNC ID, OMIM ID, RefSeq ID, protein product (with UniProt ID where applicable), known function, evidence from animal models or cell lines, associated diseases (OMIM, MONDO, ClinGen curations), phenotypes reported, established inheritance patterns, pathogenic variants, proposed mechanisms of pathogenicity, and haploinsufficiency scores (pHaplo and pLI). Genes were categorised into functional groups: protein-coding genes with known function, protein-coding genes without a described function, open reading frames (ORFs), read-through transcripts, RNA-encoding genes, and pseudogenes.

### 2.2. Clinical Findings

**Figure 1:**
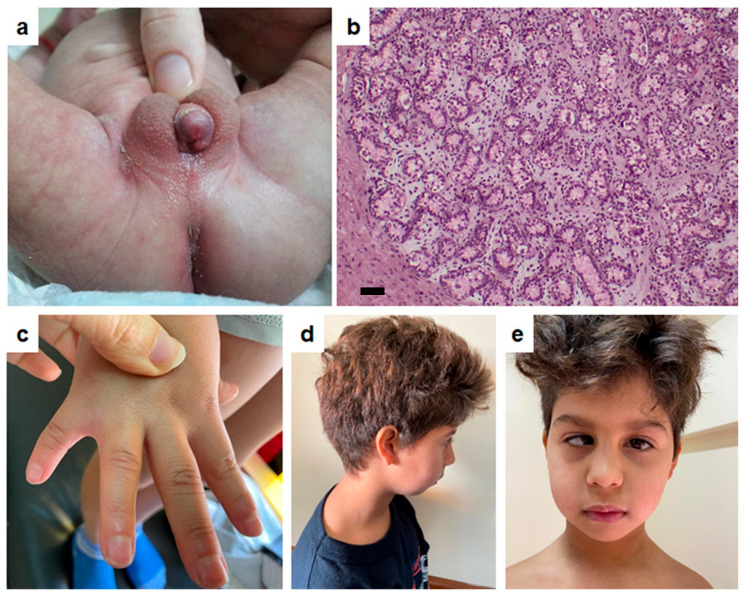
Physical and histological findings in the case reported. (**a**): external genitalia at 35 days of life. (**b**): testis histology at 7 years 6 months; solid bar = 50 µm. (**c**): clinodactyly and syndactyly. (**d**,**e**): facial dysmorphism.

The patient was a full-term baby (39 weeks) born small for gestational age (SGA) in Santiago del Estero, Argentina, and referred to our hospital for ambiguous genitalia at 1 month and 5 days of life. The baby was the first child of a self-reported non-consanguineous couple, and there was no relevant family history. Mid-parental height was 168.8 cm (25th centile). During pregnancy, mild gestational hypertension was diagnosed but did not require pharmacological treatment. Birth weight was 2.150 kg (−2.61 SDS), and length was 46.5 cm (−1.63 SDS). The patient had been hospitalised for 5 days due to respiratory distress and for nutritional recovery. Newborn screening for hypothyroidism, congenital adrenal hyperplasia, and cystic fibrosis was negative. At the first visit to our hospital, at 35 days of life, weight and length were below population standards (Table 1), and head circumference was normal. Additional positive physical findings (Figure 1) included long eyelashes, sparse eyebrows, short nose, broad nasal tip, small filtrum, retrognathia, and low-set ears. Genital examination showed a 2 cm long phallus (−2 SDS), penoscrotal hypospadias, partially fused shawl-like labioscrotal folds, and two palpable structures compatible with gonads in inguinal regions. The external genital score (EGS) was 5/12, indicating moderate undervirilisation. Abdomino-pelvic ultrasound revealed a rudimentary uterus and 2 gonads in the labioscrotal folds: right 11 × 4 × 7 mm and left 12 × 6 × 7 mm, both slightly reduced as compared to normal size. A cystourethroscopy visualised a severe hypospadic urethral meatus and a pouch compatible with a vagina. The karyotype was 46,XY [30]. Hormonal assessment at 35 days of life showed low AMH with elevated FSH and testosterone in the low-normal range with elevated LH for age and chromosomal sex. These findings led to the presumptive diagnosis of dysgenetic 46,XY DSD with syndromic features including facial dysmorphism and SGA. Male sex was assigned. During follow-up, at the age of 9 months, he had had multiple respiratory complications associated with swallowing disorders. Microcephaly, widow’s peak, and strabismus were noticed together with global developmental delay, and surgical correction of hypospadias was performed. At 2 years 6 months, there was no evidence of catch-up growth, and 5th finger clinodactyly and syndactyly of the hand together with 2–3 toe syndactyly were observed, multiple bronchoobstructive episodes had occurred, and a patent foramen ovale was diagnosed. The endocrine laboratory assessment showed normal function of thyroid and growth axes, including a normal response of growth hormone (GH) to arginine/clonidine stimulation testing, despite progressive growth failure. At 4 years 1 month, right orchidopexy was performed. At 7 years 6 months, persistent specific learning difficulties were observed. After left orchidopexy, a testicular biopsy showed hypotrophic seminiferous tubules with scarce germ cells, increased stroma, and penetration of albuginea by seminiferous tubules, indicating mild gonadal dysgenesis. Due to growth deficiency with no catch-up in a child born SGA, treatment with recombinant human growth hormone was initiated. At 9 years 1 month, myopia, attention deficit, and hyperactivity disorder were diagnosed (Figure 1). At 11 years 3 months, a slight increase in left testicular volume was observed; together with an increase in gonadotropins, they could be indicative of pubertal onset. An improvement in growth and weight was evident, in association with an elevation in serum levels of IGF1 and IGFBP3, reflecting growth hormone treatment efficacy (Table 1). In summary, the diagnosis was syndromic SGA with no catch-up growth associated with 46,XY DSD due to gonadal dysgenesis.

### 2.3. Genetic Results

**Figure 2:**
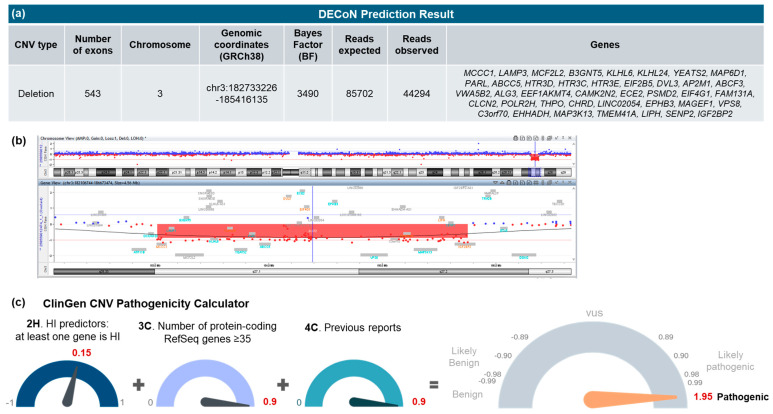
Genomic analysis of the deletion found in the patient reported. (**a**) Prediction of a heterozygous deletion in chromosome 3 based on NGS data using the DECoN algorithm. (**b**) Array-CGH plot showing the heterozygous deletion at 3q27.1q27.2, indicated by the loss of signal intensity in the corresponding probes. (**c**) Classification of the CNV according to ACMG/ClinGen criteria as pathogenic. CNV: copy number variant; HI: haploinsufficiency; NGS: next-generation sequencing.

Whole-exome sequencing (WES), which was the first diagnostic technology available in our setting, did not identify any candidate single-nucleotide variants (SNVs) in 27 genes related to 46,XY gonadal dysgenesis (*ARX*, *ATRX*, *CBX2*, *DHH*, *DHX37*, *DMRT1*, *DMRT2*, *EMX2*, *ESR2*, *FGFR2*, *GATA4*, *HHAT*, *MAMLD1*, *MAP3K1*, *MYRF*, *NR0B1*, *NR5A1*, *PPP2R3C*, *SAMD9*, *SOX9*, *SRY*, *TSPYL1*, *WNT4*, *WT1*, *WWOX*, *ZFPM2*, *ZNRF3*). Likewise, no candidate variants were prioritised through phenotype-based analysis using HPO terms across the exome, including variants predicted to have high impact. Copy number variant (CNV) analysis using DECoN predicted a heterozygous deletion on chromosome 3, spanning 543 exons. This CNV was confirmed by array-CGH, with the following result: arr[GRCh38] 3q27.1q27.2(183020090_185760128)x1, indicating a 2.7 Mb heterozygous deletion (Figure 2). The CNV spans a total of 71 genes, including 40 RefSeq genes and 35 OMIM-annotated genes, and 13 are classified as morbid genes: *ALG3*, *AP2M1*, *CLCN2*, *DVL3*, *EHHADH*, *EIF2B5*, *EIF4G1*, *IGF2BP2*, *KLHL24*, *LIPH*, *MCCC1*, *THPO*, and *YEATS2*. The microdeletion in the present case was classified as pathogenic based on ClinGen’s CNV pathogenicity calculator, which yielded a total score of 1.95, considering the following criteria: 2H (HI predictors: at least one gene is haploinsufficient = 0.15 points), 3C (number of protein-coding RefSeq genes ≥ 35 = 0.9 points), and 4C (previous reports with consistent phenotype with the genomic region but not highly specific and/or with high genetic heterogeneity = 0.9 points). This CNV was submitted to ClinVar (accession number: VCV003238952.1). The mother did not carry the microdeletion. Paternal DNA was not available.

### 2.4. Overview of 3q27.1 Microdeletion Syndrome

**Figure 3:**
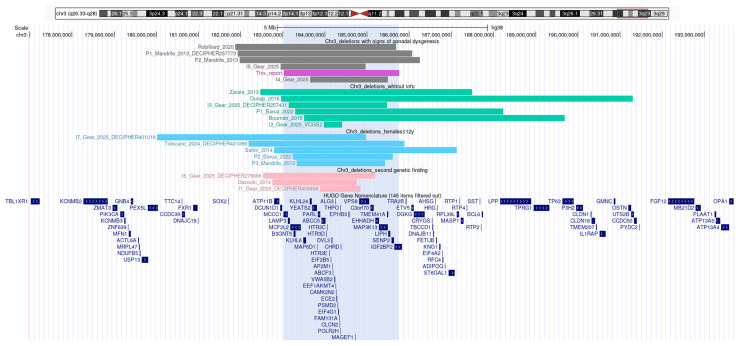
Chromosome 3 deletions described in patients with 3q27.1 deletion syndrome. Colors indicate clinical categorization: gray bars represent cases with signs of gonadal dysgenesis; green bars, cases without sufficient clinical information; blue bars, females ≤ 12 years old; pink bars, cases with a second genetic finding affecting interpretation; and the violet bar represents the present case in this study. When possible, coordinates were converted to GRCh38 using UCSC LiftOver (https://genome.ucsc.edu/cgi-bin/hgLiftOver, accessed on 9 November 2025).

SGA and short stature are prevalent features of 3q27.1 Microdeletion Syndrome (Table 2), whereas gonadal dysgenesis has not been described as a typical phenotype. However, in our systematic review of all cases reported to date [22,23,24,25,26,27,28,29,30,31,32], we identified five cases with signs compatible with mild gonadal dysgenesis (Table 2 and Supplementary Table S1): one female with premature ovarian failure (#5) and four males with cryptorchidism, micropenis, low testosterone, and/or delayed puberty (#4, 13, 19, and 20). In the remaining 16 cases, the information available was insufficient or confounding: six females were ≤12 years old, i.e., too young to suspect ovarian failure (#7, 8, 10, 12, 16, and 21); in seven cases, there was not sufficient information to rule out mild gonadal dysfunction (#2, 3, 9, 11, 14, 15, and 18); and in three cases, a second genetic finding hampered the interpretation (#1, 6, and 17) (Figure 3).

### 2.5. Gene Curation in 3q27.1 Microdeletion to Establish the DSD Genotype–Phenotype Relationship

Thirty-three protein-coding genes with known function, five protein-coding genes without a currently described function, one ORF gene, one read-through transcript, 18 genes encoding various types of RNA (none with a well-established regulatory function to date), and 13 pseudogenes were identified within the 2.7 Mb 3q27.1 microdeletion in our patient (Supplementary Table S2). *AP2M1*, *DVL3*, and *PARL* are candidate genes for prenatal and postnatal growth restriction [29,32]. On the other hand, *DVL3* and *CLCN2* are the only two genes that, according to current knowledge, could contribute to the genital anomalies. The phenotype associated with *DVL3* (i.e., autosomal dominant Robinow syndrome) includes abnormal urogenital features [33]. However, a haploinsufficiency-related mechanism, as would be the case in our patient, has not yet been reported. On the other hand, *Clcn2*-knockout mice show impairment of testicular development, leading to selective male infertility [34], but the pLI score of *CLCN2* is 0.00, indicating that the gene is tolerant to loss-of-function variants based on data available in the gnomAD population database [35].

## 3. Discussion

In this work, a 2.7 Mb deletion at 3q27.1q27.2 that had gone underdiagnosed in the first genomic assessment was identified in the reanalysis of WES data of a patient with syndromic 46,XY DSD, involving growth restriction, limb anomalies, facial dysmorphisms, and cardiac, gastrointestinal, ophthalmologic, and neurodevelopmental manifestations.

The diagnosis of 46,XY gonadal dysgenesis was established through an integrated evaluation that included clinical assessment, hormonal laboratory, abdominopelvic imaging, histological studies, and genetic testing. The combination of a 46,XY karyotype, undervirilised external genitalia, elevated gonadotropins, low AMH levels, and gonadal histology consistent with dysgenesis confirms the diagnosis.

The aetiology of 46,XY gonadal dysgenesis may involve genes that are critical specifically for testicular differentiation (e.g., *SRY*, *NR5A1*, *DHX37*) [36,37,38]. In other cases, gonadal dysgenesis occurs as part of a broader syndromic phenotype caused by alterations in pleiotropic genes that are expressed in multiple tissues during development, such as *WT1*, *SOX9*, *MYRF*, *DHH*, *PPP2R3C*, and *HHAT* [39,40]. Although most currently reported causes of 46,XY DSD are monogenic variants located in coding regions and identified through WES [38,41], CNVs involving DSD-related genes have also been described. Among them, *SOX9*, *DMRT1*, *NR5A1*, and *NR0B1* are the most frequently reported genes affected by CNVs, which have traditionally been detected using techniques such as array-CGH or MLPA [42]. In our patient, WES analysis ruled out pathogenic variants in well-known DSD-related gene exons, focusing attention on the 3q27.1 microdeletion as the potential cause of 46,XY gonadal dysgenesis.

This is the first report of gonadal dysgenesis in a 46,XY patient with a 3q27.1 microdeletion syndrome. While curated resources, such as OMIM, Orphanet, and ClinGen, provide genotype–phenotype correlations for many diseases, the 3q27.1 microdeletion syndrome is not represented in these databases. It is noteworthy that many genes and loci remain underrepresented despite clinical evidence supporting their association with DSD, as evidenced by an exhaustive overview of well-established DSD-related genes, of genes without genital phenotype reported in OMIM (e.g., *PIANP*, *FAM179B*, *TXNDC15*, *CELSR2*, *CCDC96*, and *USP2*), and of genes linked to syndromic DSD forms that are under-recognised, such as *CTU2* and *TSPYL1* [43]. One possible explanation is that patients described with 3q27.1 syndrome may have a mild genital phenotype, which may go undiagnosed unless specifically sought. Another explanation may be related to the type of CNV found in these patients. CNVs associated with human disease can be either recurrent, with a common size and breakpoint clustering, or non-recurrent, with different sizes and variable breakpoints [44]. The 3q27.1 microdeletion is a non-recurrent CNV varying in size and gene content, which makes genotype–phenotype correlations more challenging. In any case, we can rule out *SOX2*, mapping at 3q26.33, as the gene implicated in our case (Figure 3). A patient has been described with a 3q26.33 microdeletion encompassing *SOX2*, in whom syndromic DSD occurred with anophthalmia [45]. The microdeletion observed in our patient does not involve *SOX2*, thereby suggesting that other genes located within 3q27.1 may underlie the XY gonadal dysgenesis phenotype. Haploinsufficiency of dosage-sensitive genes within 3q27.1 is the proposed mechanism for this contiguous gene syndrome [29]. Our curation of the 71 genes included in the microdeletion observed in our patient identified two candidate genes potentially contributing to the DSD phenotype: *DVL3* and *CLCN2*. The hypothesis is based on their biological roles and evidence from experimental models. Interestingly, *DVL3* is also a candidate gene to explain prenatal and postnatal growth failure [29,32]. However, functional studies demonstrating that deletion of any of these genes affects gonadal development and/or linear growth would ultimately determine whether haploinsufficiency underlies the phenotypes in patients presenting with a growth failure and 46,XY DSD in association with a 3q27.1 microdeletion.

One limitation of our study is that, while the genetic evaluation included both chromosomal microarray and whole-exome sequencing, other possible genetic causes may have gone undiagnosed. These include pathogenic variants located in deep intronic or regulatory regions of known genes, which are not captured by WES, as well as balanced structural rearrangements—such as inversions or translocations—that would not be detected by array-CGH. Another limitation is that WES and array-CGH were not performed in the trio due to the unavailability of paternal samples. A negative WES result for SNVs cannot be completely conclusive without trio analysis, which is more powerful to detect de novo variants in novel genes. Although the absence of the CNV could only be confirmed in the mother, no relevant family history was evident; both parents had normal stature and no reproductive phenotype, suggesting that the condition was not inherited. This coincides with the fact that, in all cases with 3q27.1 microdeletion syndrome reported in the literature in whom the family segregation was performed, the CNV occurred as a de novo condition.

## 4. Conclusions

In conclusion, although the 3q27.1 microdeletion provides a compelling syndromic framework for many of the features observed in our patient, the gonadal dysgenesis may represent a previously underreported phenotype within this spectrum. Functional studies of the proposed candidate genes could clarify whether haploinsufficiency contributes to any of the features associated with this non-recurrent deletion, helping to establish more precise genotype–phenotype correlations, guide comprehensive assessments of affected organ systems, and support individualised clinical follow-up.

## Figures and Tables

**Table 1:** Clinical features of the index case during follow-up.

Age	35 Days	9 Months	2 Years 6 Months	4 Years 1 Month	7 Years 6 Months	9 Years 1 Month	11 Years 3 Months
Length/stature (SDS)	49 cm (−2.6)	66 cm (−2.4)	80 cm (−2.9)	87 cm (−3.5)	102 cm (−4.1)	108 cm (−3.9)	121 cm (−3.0)
Weight (SDS)	2.75 kg (−3.0)	6.85 kg (−2.3)	9.25 kg (−2.5)	10.9 kg (−4.3)	15.0 kg (−2.0)	18.4 kg (−2.9)	27.9 kg (−1.6)
Dysmorphisms	Facial	No changes	Fingers and toes	No changes	No changes	No changes	No changes
Urinary and genital findings	Phallus 2 cm, small inguinal gonads, ambiguous genitalia, rudimentary uterus and vaginal pouch	No changes	No changes	No changes	G1, PH1, phallus 2.5 cm, RT 2 cc, LT inguinal	G1, PH1, RT 2 cc, LT 2 cc, both scrotal	G1, PH1, RT 3 cc, LT 2 cc, both scrotal
Neurodevelopment	Normal	Global developmental delay	No changes	No changes	Intellectual disability	ADHD	No changes
Other findings	Head circumference 35.5 cm (3rd centile)	Microcephaly; widow’s peak, strabismus; swallowing disorder and respiratory complications	Foramen ovale Bronchoobstructive episodes	No changes	No changes	Myopia	No changes
Laboratory findings	Karyotype 46,XY T 206 ng/dL (180–270) AMH 417 pmol/L (421–1470) LH 8.2 IU/L (0.2–4.2) FSH 6.7 IU/L (0.3–4.7)	NA	T < 10 ng/dL (<10) AMH 404 pmol/L (236–1831) LH < 0.1 IU/L (<0.1–0.3) FSH 0.8 IU/L (0.3–1.7) TSH 4.9 mIU/L (0.5–6.5) fT4 1.05 ng/Dl (0.8–2.2) IGF1 66 ng/mL (29–118) IGFBP3 3.5 mg/L (1.7–4.2) GH peak 24.8 ng/mL (>4.8)	NA	T <10 ng/dL (<10) AMH 240 pmol/L (236–1831) LH < 0.1 IU/L (<0.1–0.3) FSH 1.4 IU/L (0.3–1.7) TSH 2.6 mIU/L (0.5–6.5) fT4 1.04 ng/dL (0.8–2.2) IGF1 92 ng/mL (39–132) IGFBP3 4.7 mg/L (2.0–4.4)	NA	T < 10 ng/dL (<10) AMH 107 pmol/L (236–1831) LH 1.8 IU/L (<0.1–0.3) FSH 8.3 IU/L (0.3–1.7) TSH 4.6 mIU/L (0.5–6.5) fT4 1.14 ng/dL (0.8–2.2) IGF1 214 ng/mL (47–268) IGFBP3 5.8 mg/L (2.2–5.6)
Treatment		Hyspospadias correction		Right orchiopexy	Left orchiopexy and biopsy: testicular dysgenesis	rhGH 0.35 mg/kg/week	rhGH 0.35 mg/kg/week

**Table 2:** Clinical and genetic characteristics of the present case and overview of all patients with 3q27.1 microdeletion syndrome previously reported in the literature.

	This Report	Overview
Chromosomal regions (GRCh38)	3q27.1q27.2 (chr3:183,020,090–185,760,128)	SRO 3q27.1 (chr3:183,978,599–184,406,506)
Size of deletion	2.71 Mb	0.4–8.4 Mb
Inheritance	Unknown	14 de novo 8 unknown
Sex	Male	13 Female 9 Male
Age at last examination	11 years	Foetus—25 years
Oligohydramnios	No	5/22
Small for gestational age	Yes	21/22
Short stature	Yes	15/22
Microcephaly	Yes	17/22
Facial dysmorphisms	Low-set ears, retrognathia, widow’s peak, flat forehead, highly arched eyebrows, long eyelashes, upslanted palpebral fissures, broad nasal tip, smooth philtrum, prominent nasal bridge, thick vermilion border	21/22
Eye defects	Bilateral convergent strabismus; myopia	10/22
Hearing loss	No	3/22
Dental abnormalities	No	7/22
Hand abnormalities	5th finger clinodactyly and syndactyly	12/22
Feet abnormalities	2–3 toe syndactyly	10/22
Heart defects	Patent foramen ovale	10/22
Respiratory problems	Respiratory distress, recurrent respiratory infections	10/22
Thrombocytopenia	No	5/22
Urogenital abnormalities	Penis 2 cm (−2 SDS), penoscrotal hypospadias, partially fused labioscrotal folds and inguinal gonads. EGS 5/12.	8/22
Hypotonia	No	11/22
Cognitive abnormalities	Specific learning disability	19/22
Behaviour disorders	Attention deficit hyperactivity disorder	8/22
Feeding disorders	Dysphagia	14/22
Seizure	No	4/22
Additional genetic findings	No	2/22

## Data Availability

The datasets generated during and/or analysed during the current study are available in the Sequence Read Archive (SRA) repository. The names of the repository and accession number can be found at NCBI PRJNA1055058. https://www.ncbi.nlm.nih.gov/bioproject/PRJNA1055058/ (accessed on 15 September 2025).

## Supplementary Materials

The following supporting information can be downloaded at: https://www.mdpi.com/article/10.3390/ijms27020821/s1.
